# Promising *Aedes aegypti* Repellent Chemotypes Identified through Integrated QSAR, Virtual Screening, Synthesis, and Bioassay

**DOI:** 10.1371/journal.pone.0064547

**Published:** 2013-09-06

**Authors:** Polina V. Oliferenko, Alexander A. Oliferenko, Gennadiy I. Poda, Dmitry I. Osolodkin, Girinath G. Pillai, Ulrich R. Bernier, Maia Tsikolia, Natasha M. Agramonte, Gary G. Clark, Kenneth J. Linthicum, Alan R. Katritzky

**Affiliations:** 1 Department of Chemistry, University of Florida, Gainesville, Florida, United States of America; 2 Medicinal Chemistry Platform, Ontario Institute for Cancer Research, Toronto, Ontario, Canada; 3 Department of Chemistry, Lomonosov Moscow State University, Moscow, Russia; 4 Institute of Chemistry, University of Tartu, Tartu, Estonia; 5 USDA-ARS-CMAVE, Gainesville, Florida, USA; 6 Chemistry Department, King Abdulaziz University, Jeddah, Saudi Arabia; Universidade Federal do Rio de Janeiro, Brazil

## Abstract

Molecular field topology analysis, scaffold hopping, and molecular docking were used as complementary computational tools for the design of repellents for *Aedes aegypti*, the insect vector for yellow fever, chikungunya, and dengue fever. A large number of analogues were evaluated by virtual screening with Glide molecular docking software. This produced several dozen hits that were either synthesized or procured from commercial sources. Analysis of these compounds by a repellent bioassay resulted in a few highly active chemicals (in terms of minimum effective dosage) as viable candidates for further hit-to-lead and lead optimization effort.

## Introduction

**Figure 1:**
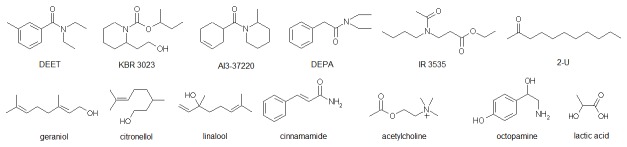
Various synthetic and natural insect repellents and attractants.

Natural sources, such as local herbs and gum, oil and plant-based smoke, have been used by mankind for millennia as mosquito repellents and are still utilized today by 50-90% of residents throughout the rural tropics [1]. Intensive research to discover more effective, long-lasting, and water-resistant repellents began during WWII because of more than one million cases of malaria recorded among the U.S. troops involved in overseas campaigns [1]. The most effective wide-spectrum synthetic repellent to emerge from this program was N,N-diethyl-3-methylbenzamide (DEET) (see Figure 1) discovered in 1952.

Although considered a gold standard for insect repellents, DEET does have disadvantages: (i) limited efficacy against *Anopheles* *albimanus* (the principal malaria vector in Central America and the Caribbean) [2], tolerant varieties of *Aedes aegypti* [3], and some other vectors [4] (ii) skin irritation; (iii) possible neurotoxicity [5]; (iv) a plasticising action on polymeric materials; and (v) relatively high cost. Additional repellent active ingredients (Figure 1) such as the piperidine derivatives KBR 3023 (picaridin) and AI3-37220 are considered almost as efficacious as DEET, and in some cases reported to remain effective for a longer duration and have more desirable cosmetic properties. The repellent diethyl phenylacetamide (DEPA) is as reported to be as efficacious as DEET and can be produced at about half the cost of DEET. The ethyl ester of 3-[N-butyl-N-acetyl]-aminopropionic acid (IR3535), although less efficacious than DEET, is favored by some consumers because of a low incidence of side effects since its development in 1975. The naturally and synthetically available compound 2-undecanone (2-U) was recently reported as a repellent against mosquitoes and ticks [6,7,8].

Computational studies of mosquito repellency have been attempted far less frequently than for drug discovery. Since the discovery of DEET, many experimental efforts have been devoted to finding a superior repellent and some of those consisted of evaluation of DEET analogues and other structurally similar carboxamides. One QSAR (Quantitative Structure-Activity Relationships) pharmacophore model predicted the most favorable amide structure to consist of an aliphatic moiety and an aromatic hydrophobic moiety separated by a highly polar carboxyl group [9]. Another 3D (three-dimensional) QSAR model defined an optimal structural pattern that consists of two oxygen atoms (one of which belongs to an amide group) positioned a certain distance from each other and joined by a lipophilic moiety [10]. Predictive models have also been derived by using multi-linear QSAR based on experimental [11] and theoretical [12] descriptors. Protection times of a large set of carboxamides and N-acylpiperidines were qualitatively analyzed using artificial neural networks and multiple linear regression [13,14]. One more example is the study of sesquiterpenes occurring in essential oils of plants that possess remarkable insect repellent ability, sometimes comparable in efficacy to DEET [15]. The repellents in this study were classified as early spatial, late spatial, and contact. It was also stressed that a few chemical bond separation between the hydroxyl and the hydrophobic fragments is beneficial for repellent activity. All of the above computational studies were based solely on structural characteristics of odorants. Until very recently, no valid information on putative molecular targets was available.

### 1 Mechanism of action

Despite an increase in research effort over the last several decades, the mechanism of repellency is not yet fully understood. According to the known modes of action, chemicals affecting insects are classified as controlling (i) growth and development, (ii) energy metabolism, (iii) nerves and muscles. Since contact repellents are fast acting agents, their mechanism of action is more likely to be due to the last of these three types, which may include inhibition of acetylcholinesterase (AChE), modulation of sodium channels, and modulation of nicotinic acetylcholine receptors. A highly probable mechanism for repellency is the interference with the insect chemosensory system that governs behavioral patterns such as host-seeking, oviposition, and fleeing from chemical irritants. For example, DEET is evidenced to modulate olfaction in insects [16], inhibit acetylcholinesterase activity [4,17] and affect gustatory receptors [18].

#### 1.1 Olfactory system as the target

The insect olfactory system is believed to be the prime target for many natural repellents. Olfactory transduction in insects can be subdivided into three successive stages: (i) encoding of a stimulus caused by an odorant into a neuronal signal, (ii) decoding of the signal in the antennal lobe, and (iii) association with perceptual qualities in higher CNS centers. Normally an odorant penetrates through the pores in the sensillum cuticle to the hemolymph, which bathes dendrites of the olfactory receptor neurons (ORN). Odorant binding proteins (OBPs) also present in the hemolymph recognize and encapsulate hydrophobic odorants for further transportation through the hemolymph to specialized odorant receptors (ORs) residing in the ORN membrane [2,19,20,21]. Stimulation of an odorant receptor by an odorant initiates a sequence of biochemical events amplifying the action potential [22]. One odorant can elicit responses of different intensities from different ORs, whereas ORs can be broadly or narrowly tuned for a wide or restricted panel of odors [23,24]. The odor code reflects not only the odorant chemical structural structure but also depends on its concentration [25,26] and presence of other volatiles [27]. A systematic analysis of a large collection of odor responses by the odorant receptor body revealed hundreds of OR combinations building up a multi-dimensional odor space, which characterises an odor with respect to OR and vice versa, and also provided valuable statistics on excitatory and inhibitory responses [22,23]. Several more models were proposed to reproduce various stages of the odorant transduction cascade: (i) multi-step biophysical models of single ORNs for vertebrates [28,29] and insects [30,31,32], including a statistical distribution of collective firing rates [33] (ii) use of artificial neural networks and molecular parameters for the prediction of responses in *Drosophila melanogaster* [34]; or (iii) mimicking all three stages of the olfaction process [35].

#### 1.2 *Aaeg*OBP1 as a molecular target

The yellow fever mosquito, *Aedes aegypti*, has 66 identified odorant binding proteins [36], while more than 80 OBP encoding genes were found in the *Anopheles gambiae* genome [37]. Recently published studies of OBP are based on available crystallographic data, biochemical assays, and *in silico* molecular modeling and docking. For example, ligand affinities of some benzoates and phthalates were experimentally measured for *Aaeg*OBP22 using immunofluorescence and fluorescent probe techniques [35,38]. X-Ray structures were resolved for several mosquito OBPs, among them *Agam*OBP07 complexed with 4-hydroxy-4'isopropyl-azobenzene and palmitic acid [39], *Agam*OBP22a complexed with benzaldehyde, glycerol, and cyclohexanone [40], *Agam*OBP4 complexed with indole [41], and *Agam*OBP47 [42]. Highly abundant in mosquitoes, OBP1 is the most probable candidate for the host-seeking and oviposition behavior, as it is overly expressed in the female antennae, not in the male ones [43, 44]. X-Ray structures were resolved and analyzed for orthologous OBP1 in *Anopheles gambiae* (*Agam*OBP1, complexed with polyethelyneglycol, PEG [45] and DEET [46]), *Culex* *quinquefasciatus* (*Cqui*OBP1, complexed with the oviposition pheromone (5R,6S)-6-acetoxy-5-hexadecanolide, MOP [47]), and *Aedes aegypti* (*Aaeg*OBP1, complexed with PEG [48]) sharing as high as 87-90% of the sequence identity. Electroantennogram responses and *in vitro* binding assays revealed that *Agam*OBP1, *Cqui*OBP1, and *Afun*OBP1 had high binding affinities towards indole, 1-octen-3-ol (human skin effluents) as well as towards geranylacetone, octanal, 2-U, and some other elongated hydrophobic molecules. Taken together these studies suggest that *Aaeg*OBP1 can be a promising candidate for structure-based design of mosquito repellents.

Interaction of odorants with OBPs and ORs and ORN responses are potentially attractive targets for QSAR analysis: expressed quantitatively they can be used as predictors for repellency or attraction along with theoretical and empirical molecular descriptors of natural and synthetic semiochemicals.

OBPs represent the first “selection gate” in a multistage odor perception process. Expression, purification, and crystallization are currently becoming possible for OBPs which has made the tertiary structures of some OBPs available for computer modeling [45]. However, biological systems feasible for efficient, large-scale productions of ORs have been proposed only recently [49,50], and partial homology modeling and docking studies have been performed so far only for human G protein-coupled ORs [51]. As for the neurophysiological responses, they can be quantified as currents, spike frequencies of a specific ORN, or activity changes within a set of glomeruli of the antennal lobe. The latter, for example, was recently studied along with directed and undirected movement responses for attractive odors in walking *Drosophila* [52]. In mosquitoes, ORN currents were studied for several repellents (DEET, 2-U, S220, callicarpenal, pyrethroid) in *Aedes aegypti* [5, 53] and for a 110-odorant panel in *Anopheles gambiae* [54]. Such an increase in the amount of available electrophysiological data hopefully brings closer the opportunity to directly correlate electrophysiology and behavioral data.

The search for more stable and potent repellents that are less toxic to humans and are environmentally benign is of imminent importance. Mosquitoes continue to be vectors that cause diseases such as malaria, West Nile virus, yellow fever, among others of medical and veterinary significance. An ideal repellent needs to be highly effective and long-lasting, while nontoxic for humans and other non-target species. It also has needs user acceptance, implying that it has benign or desirable cosmetic characteristics. Another very important issue is the cost of production and deployment, because much of the malaria threat resides in Africa and many African nations cannot afford expensive vector control tools. An integrated computational approach would be highly relevant in this regard. It can shorten discovery time and lower cost by reduction of the vast resources required for classical trial-and-error methods based on screening of large compound libraries.

**Table 1:** Experimental and predicted by the MFTA model *Ae*. aegypti repellency for 43 carboxamides and DEET^**a**^.

**ID**	**Name**	**MED_obs_.**	**MED_pred_.**	**ID**	**Name**	**MED_obs_.**	**MED_pred_.**
**5a**	*N*-butyl-*N*-methyl-hexanamide	0.117	0.147	**5f'**	(E)-*N*,*N*-di-(2-methylpropyl)-2-hexenamide	0.625	0.600
**5b**	*N*-butyl-*N*-ethylhexanamide	0.156	0.160	**5f**	*N-*ethyl-*N-*phenylhexanamide	0.625	0.257
**5c**	*N*,*N*-diallylhexanamide	0.195	0.290	**5m**	*N*-cyclohexyl-*N*-ethyl-3-methylbutanamide	0.172	0.211
**5g**	*N-*butyl-*N-*ethyl-2-methylpentanamide	0.104	0.132	**5q**	*N-*butyl-*N*-ethyl-2-methylbenzamide	0.156	0.176
**5i**	*N*-butyl-*N*,2-diethylbutanamide	0.125	0.155	**5v**	*N*-ethyl-2-methyl-*N*-(2-methyl-2-propenyl)benzamide	0.145	0.093
**5j**	*N*,2-diethyl-*N*-(2-methyl-2-propenyl)butanimide	0.375	0.306	**5w**	*N*-ethyl-2-methyl-*N*-phenylbenzamide	5.160	7.012
**5k**	*N*-butyl-*N*-ethyl-3-methylbutanamide	0.125	0.164	**5k'**	*N-*cyclohexyl-*N-*methylheptanamide	0.172	0.127
**5l**	*N*,*N*-diisobutyl-3-methylbutanamide	0.406	0.305	**5l'**	(E)-*N-*cyclohexyl-*N*-ethyl-2-methylpent-2-enamide	0.140	0.153
**5n**	*N*-butyl-*N*-ethyl-2,2-dimethylpropanamide	0.286	0.260	**5d**	Hexahydro-1-(1-oxohexyl)-1*H*-azepine	0.033	0.108
**5o**	*N*-ethyl-2,2-dimethyl-*N*-(2-methyl-2-propenyl)propanamide	0.469	0.512	**5h**	1-(1-azepanyl)-2-methyl-1-pentanone	0.102	0.089
**5p**	1-(1-azepanyl)-2,2-dimethyl-1-propanone	0.313	0.284	**5t**	(E)-1-(1-azepanyl)-2-methyl-2-penten-1-one	0.098	0.089
**5r**	(E)-*N-*butyl-*N*-ethyl-2-methyl-2-pentenamide	0.117	0.119	**5a'**	hexahydro-1-(3-methylcrotonoyl)-1*H*-azepine	0.140	0.109
**5s**	(E)-*N*-ethyl-2-methyl-*N*-(2-methyl-2-propenyl)-2-pentenamide	0.182	0.234	**5b'**	*N*-butyl-*N*-ethyl-cinnamamide	10.750	15.268
**5u**	(E)-2-methyl-*N*,*N*-di-2-propenyl-2-pentenamide	0.417	0.216	**5c'**	*N*,*N*-bis(2-methylpropyl)-3-phenyl-2-propenamide	20.125	28.443
**5x**	*N*-butyl-*N*-ethyl-3-methyl-2-butenamide	0.192	0.145	**5d'**	*N*-ethyl-*N*,3-diphenyl-2-propenamide	20.250	24.890
**5y**	*N*-ethyl-3-methyl-*N*-(2-methyl-2-propenyl)-2-butenamide	0.313	0.285	**5i'**	*N*,3-dicyclohexyl-*N*-ethylpropanamide	20.500	24.830
**5z**	*N*,*N*-diisobutyl-3-methylcrotonamide	0.219	0.269	**C39**	3-cyclohexyl-N-methyl-N-octylpropanamide	25.000	31.596
**5e'**	(E)-*N*-n-butyl-*N*-ethyl-2-hexenamide	0.274	0.322	**C40**	4-methyl-N-phenylbenzamide	25.000	14.768
**5g'**	(E)-*N*-cyclohexyl-*N*-ethyl-2-hexenamide	0.651	0.414	**C41**	2-methyl-N-phenylbenzamide	25.000	17.736
**5h'**	*N*-butyl-*N*-methyl-5-hexynamide	0.182	0.203	**C42**	N-cyclohexyl-N-isopropyl-4-methyloctanamide	25.000	28.247
**5j'**	(E)*-N*,2-dimethyl-*N*-octylpent-2-enamide	0.125	0.195	**C43**	N,N-dicyclohexyl-4-methyloctanamide	25.000	20.564
**5e**	*N-*cyclohexyl-*N-*ethylhexanamide	0.266	0.205	**DEET**	N,N-diethyl-3-methylbenzamide	0.052	0.053

a MED stands for the minimum effective dosage, µmol/cm^2^

In this manuscript we report a fundamental effort integrating computational and experimental approaches to the design of novel mosquito repellents. Modern molecular modeling techniques such as QSAR and molecular docking are integrated for the first time with experimental bioassay to guide rational design of mosquito repellents in a way similar to that widely used in drug discovery. The potential of the proposed integrated approach first tested on *Aedes aegypti* repellents will be applied in the future to the development of a broader class of vector control systems.

## Materials and Methods

### 1: Ethics Statement

All volunteers provided written informed consent to participate. A total of three male and two female volunteers, ages 35, 36, and 44, 26, and 44, respectively, participated in this study; each volunteer tested each compound once until a consecutive pass/fail result had been achieved (this consisted of one replicate). The protocol was approved by the University of Florida Human Use Institutional Review Board (IRB)-01 as protocol 636-2005. The approval has been renewed annually since 2005.

### 2: Dataset

**Figure 2:**
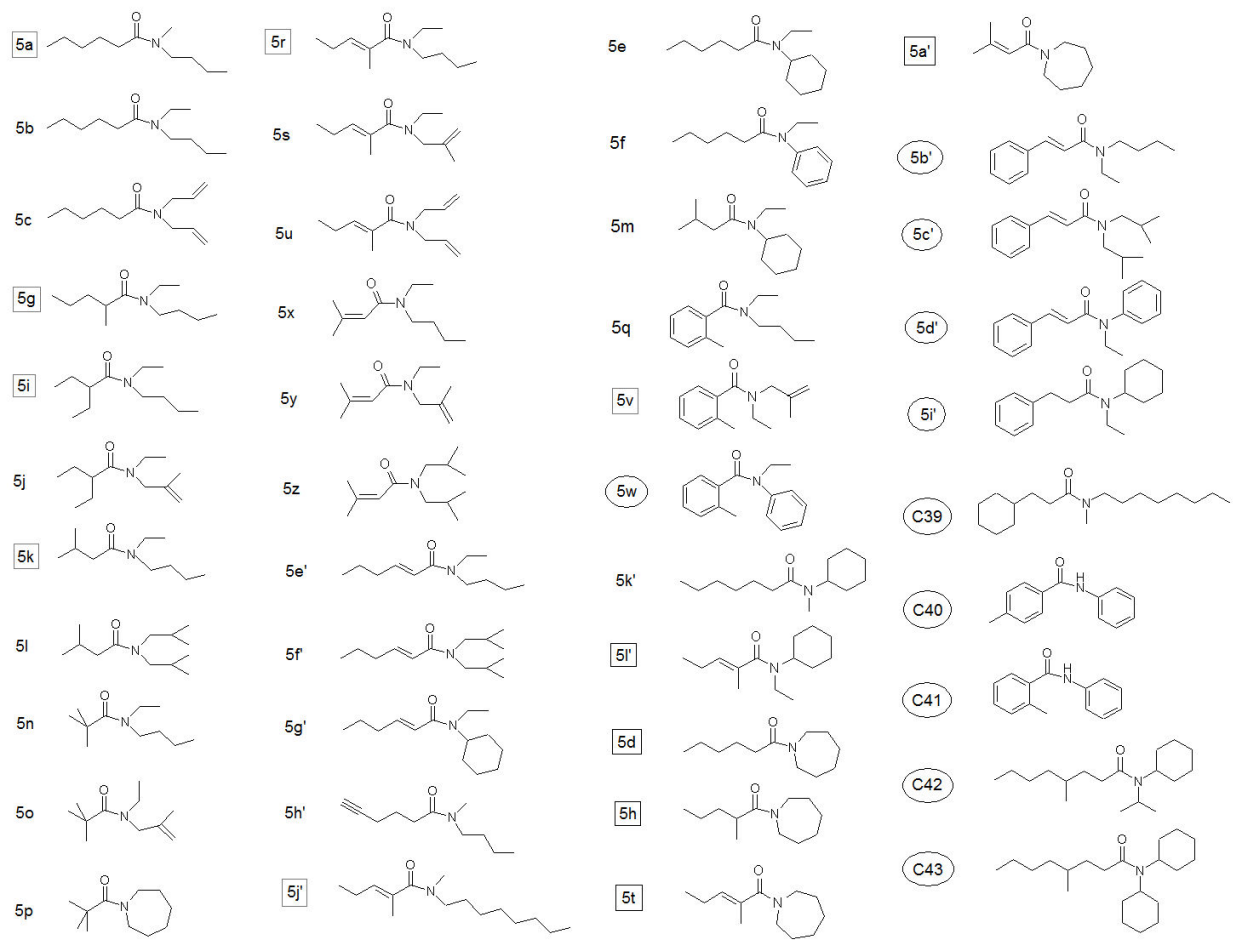
Chemical structures of 43 carboxamides. The most active compounds, with MED < 0.150 µmol/cm^2^, are marked with squares; the least active compounds, with MED > 5 µmol/cm^2^, are marked with circles.

**Figure 3:**
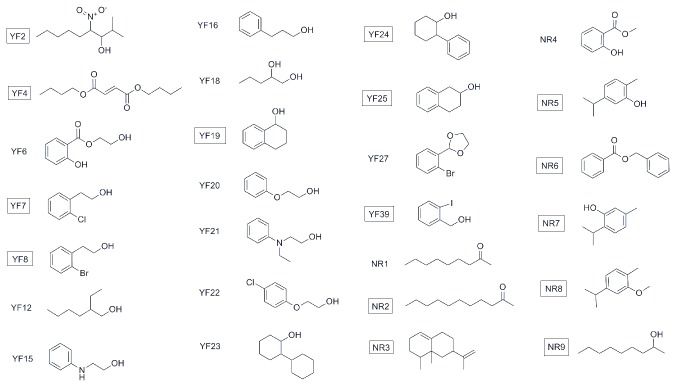
Chemical structures of 27 assorted compounds. The most active compounds are marked with squares.

The training set for QSAR analysis consisted of 43 carboxamides published previously by our group [13] together with 27 compounds for which the repellency was evaluated for this study. The previously published carboxamides were proposed for synthesis and biological testing on the basis of results from a neural network classification model [29], while the 27 additional compounds are selected from USDA internal records. In this study, repellent activity was characterised by a minimum effective dosage (MED, µmol/cm^2^), which is the measurement of the minimum surface concentration of a compound that is required to produce a repellent effect. Based on the selected endpoint for this study, it is an estimate of the effective dose for 99% repellency (ED_99_) because the failure threshold is selected to be 1% (5 bites out of approximately 500 mosquitoes in 1 min). For QSAR analysis purposes, logarithmic values of MED were used in order to relate the values to changes of the free energy. The training set structures and respective MED values are given in Figures 2-3 and Tables 1-2, respectively. Compounds **5a** through **C43** are the carboxamides, while structures **YF2** to **YF39** (YF means yellow fever) are the 27 assorted compounds containing hydroxyl, ether, ester, amine, nitro, and halogen functionalities. The additional natural compounds (**NR1** to **NR9**) are derived from studies of the plant genus *Hedychium* .

**Table 2:** Experimental and predicted by the MFTA model *A. aegypti* repellency for 27 assorted compounds.

**ID**	**Name**	**MED_obs_.**	**MED_pred_.**	**ID**	**Name**	**MED_obs_.**	**MED_pred_.**
**YF2**	2-methyl-4-nitro-3-nonanol	0.047	0.043	**YF24**	2-phenyl-cyclohexanol	0.047	0.058
**YF4**	dibutyl fumarate	0.047	0.036	**YF25**	1,2,3,4-tetrahydro-2-naphthol	0.062	0.078
**YF6**	2-hydroxyethyl 2-hydroxybenzoate	2.500	2.716	**YF27**	2-(2-bromophenyl)-1,3-dioxolane	0.156	0.169
**YF7**	2-chlorophenethyl alcohol	0.101	0.078	**YF39**	(2-iodophenyl)methanol	0.070	0.066
**YF8**	2-bromophenethyl alcohol	0.049	0.076	**NR1**	2-nonanone	0.437	0.285
**YF12**	2-ethyl-1-hexanol	1.875	1.351	**NR2**	2-undecanone	0.109	0.086
**YF15**	2-anilinoethanol	1.875	0.679	**NR3**	valencene	0.138	0.157
**YF16**	3-phenyl-1-propanol	0.406	0.531	**NR4**	methyl salicylate	0.312	0.278
**YF18**	1,2-pentanediol	2.500	2.458	**NR5**	carvacrol	0.013	0.013
**YF19**	1,2,3,4-tetrahydro-1-naphthol	0.078	0.068	**NR6**	benzyl benzoate	0.023	0.071
**YF20**	2-phenoxyethanol	0.563	0.578	**NR7**	thymol	0.031	0.050
**YF21**	2-(N-ethylanilino)-ethanol	0.156	0.113	**NR8**	carvacrol methyl ether	0.063	0.050
**YF22**	2-(*p*-chlorophenoxy)-ethanol	0.219	0.240	**NR9**	2-nonanol	0.066	0.084
**YF23**	2-cyclohexyl-cyclohexanol	0.437	0.513				

### 3: Molecular Field Topology Analysis, MFTA

MFTA is an analytical tool that provides analysis of quantitative structure-activity relationships of structurally related compounds. One can consider it as a topological analogue of CoMFA [55]: instead of spatial alignment of structures in a three-dimensional grid, 2D (two-dimensional) molecular graphs are superimposed to make the so-called “molecular supergraph” abbreviated as MSG [56]. The molecular graph is a useful mathematical abstraction widely used by chemists; see for example a recent review by Pogliani and co-authors [57]. Simply put, it is just a structural formula where atoms are connected by bonds. The MSG vertices and edges corresponding to atoms and bonds are furnished with values of local atomic descriptors to form a rectangular atom-descriptor matrix which is processed by the partial least squares method to tie experimental activity to chemical structure. The basic MFTA descriptor pool includes the following molecular fields: atomic charges, van der Waals radii, electronegativity, hydrogen bond parameters, and lipophilicity, although more fields can be invoked. Construction of the MSG is controlled by a number of settings, such as allowing mapping of cyclic to acyclic fragments, matching of atoms and bonds according to their type, label, charge, multiplicity, etc., and even forcing the superposition of certain mismatching functional groups. Predictive quality of a model is characterised by the squared correlation coefficient, *R* ^2^, and the cross-validation coefficient *Q*_*n*_^2^, where *n* is a user-defined parameter for the number of structures in each leave-many-out cross-validation run. MFTA has been successfully applied to several medicinal chemistry problems such as (i) discovery of new C-X-C chemokine receptor-4 antagonists [58], (ii) modeling of anticholinesterase activity of o-phosphorylated oximes [59], and (iii) design of GABA_A_ receptor selective ligands [60].

### 4: Hit Expansion and Molecular docking

Hit expansion was performed based on the structures of the four most potent repellents: DEET, picaridin (KBR 3023), **5d** (Table 1) and **YF23** (Table 2). The 2D structures of the molecules were sketched with ChemDraw and saved in the MOL format (a widely accepted text file format for storing atomic coordinates and chemical information) for further processing. We selected 29 structures to conduct substructure and similarity searches against the eMolecules Plus database of approximately 5 million unique commercially available compounds [61] using Pipeline Pilot 8.0 from Accelrys. Similarity searches were conducted with the 0.65 Tanimoto similarity cutoff based on FCFP-4 Pipeline Pilot fingerprints. After applying OICR lead-like filters and molecular weight cutoff of 250 Da, the search resulted in 47 analogues of DEET, 30 analogues of picaridin, 59 analogues of **YF23**, and 208 analogues of **5d**. To evaluate this large number of compounds and do a more rational selection of candidates for experimental testing, we docked all the 344 virtual hits (including the original four lead compounds) in the A monomer of the *Aedes aegypti* OBP1 structure (PDB 3K1E, 1.85 Å) [47]. Despite the fact that the *Anopheles gambiae* OBP1 structure (PDB 3N7H, 1.6 Å) [45] was solved at a higher resolution, we selected the *Aedes aegypti* 3K1E structure for docking because of our interest in this particular mosquito species. These OBP1 proteins both share high sequence identity (75.9% as calculated by the NEEDLE pairwise sequence alignment EBI tool, http://www.ebi.ac.uk/Tools/) and nearly identical 3D structures (RMSD of 0.16 Å and 0.25 Å based on backbone and all heavy atom superposition, correspondingly).

We docked all the scaffold hopping hits as well as compounds represented in Tables 1 and 2 in the OPB1 3K1E structure with the Glide software (Schrödinger Inc.) using the SP and XP scoring functions (G1, G2). Geometries of the ligands were converted from 2D to 3D with LigPrep (Schrödinger Inc.) using the OPLS2005 force field, and then the structures were ionized with Epik to pH 7.0 and finally energy minimized with default LigPrep settings. Defined chiral centers were preserved, while all undefined chiral centers were enumerated. The receptor protein GRID was calculated for a large box that was defined in the presence of the nine-unit polyethylene glycol (PEG) molecule that protrudes the whole inner channel inside of the OBP1 protein of approximately 27 Å in length. A standard box size of 10 Å beyond from each side from the ligand was used. Neither positional nor hydrogen bonding constraints were applied. The hydroxyl of Tyr122 was designated as a rotatable group. For the docking stage we selected the following options: sampling ring conformations (also include the initial ring conformation) and nitrogen inversions, penalizing non-planar conformations of amides and adding Epik state penalties to the docking score. We used the default scaling of van der Waals radii by 0.8 for atoms with partial atomic changes less than 0.15. We generated up to one million poses during the docking run, and then selected 100 best poses for post-docking minimization and saved top five poses for each ligand. We repeated the same docking procedure using the XP scoring function. However, due to the cross-docking nature of this calculation (ligands are docked in the protein structures that were not solved with any of these ligands), we obtained more consistent results with the SP scoring function. The docking poses were carefully inspected for the presence of strong hydrogen bonding and van der Waals interactions as well as steric clashes. Compounds that fit the best in the OBP1 structure were selected for ordering.

### 5: Synthesis

2-Methyl-4-nitrononan-3-ol (**YF2**) was obtained in a 36% yield by the treatment of 1-nitrohexane with isobutyraldehyde and DBU in acetonitrile. Compounds **5a** to **5k** were synthesized according to the previously published protocols [13,14].

The rest of the hit expansion compounds were purchased from Aldrich (**DEET**, **YF4**), Sigma (**YF7**, **YF8, YF19**, **YF24**, **YF28** and **YF39**), TCI America (**YF12**-**YF18**, **YF20**-**YF23**), Acros Organics (**YF25**), VWR International (**YF27**), and Oakwood (**YF40**).

### 6: Bioassay

The test mosquitoes were female *Ae* *. aegypti* (Orlando strain, 1952) from the colony maintained at United States Department of Agriculture-Agricultural Research Service-Center for Medical, Agricultural and Veterinary Entomology (USDA-ARS-CMAVE) in Gainesville, FL. Pupae were obtained from the colony and newly emerged mosquitoes were maintained on 10% sugar water and kept in laboratory cages at an ambient temperature of 28 ±1 ^o^C and RH of 35-60%. Host seeking behavior was pre-selected in nulliparous female mosquitoes aged 6-10 days, indicated by flight upwind towards a potential host, from stock cages using a hand-draw box and trapped in a collection trap [62]. After 500 (± 10%) females were collected in the trap, they were transferred to a test cage (approximately 59,000 cm^3^ with dimensions 45 x 37.5 x 35 cm) and allowed to acclimatize for 17.5 (± 2.5) min before testing was initiated.

Just prior to the experiment, the pieces of treated cloth are removed from the vials and stapled onto card stock tabs (5 x 3cm). Each piece of the cards and the assembly were hung on a drying rack using masking tape for 3-5 min. Participants in the study used latex gloves to pull a nylon stocking over their arm. A Velcro™-sealed vinyl sleeve was then placed over the forearm. The sleeve had a 32-cm^2^ (4 x 8cm) window to allow attractive skin odors to escape and draw mosquitoes to that open area. The purpose of the nylon stocking was to produce a barrier between the dried cloth and the skin, thereby avoiding direct contact of chemical to skin. The dried cloth assembly was affixed over the opening in the sleeve and held in place with masking tape. Participants then inserted their arm with the sleeve and patch into a screened cage that contained female *Ae* *. aegypti* mosquitoes. Tests were conducted on each control or treated patch for 1 min. A control patch (acetone solvent only, then dried) was tested prior to the start of experiments and evaluation of the same untreated control patch after every 10 tests. If five landings were not received on the control patch in 30 s, then tests were discontinued for 60 min. At the conclusion of testing the control patch was tested again. If five landings were not received within 30 s, the data for the replicate was discarded. When testing a patch treated with a candidate repellent, if approximately 1% or 5 mosquito bites were received during this one minute test, this compound was considered to have failed, i.e. was not repellent at that concentration. If a treated cloth patch received 0-4 bites within a min, then it was considered as passed, i.e., repellent at that concentration of the test compound. The median concentration patch was tested in the first round and treated patches were then tested successively at higher or lower concentrations depending upon whether the previous patch failed or passed, respectively.

The time interval between each tested patch was < 90 seconds until 10 successive tests had been conducted. If appreciable mortality had occurred during testing, the number of knocked down mosquitoes are estimated and additional female mosquitoes are added to the cage to keep the available mosquitoes at approximately 500. The estimate of the MED was the lowest concentration that passed for each candidate. Observed MED values for each candidate compound were averaged across participants and reported as a mean MED ± standard error. Additional explanation of this type of bioassay can be found elsewhere [13,14,63,64].

## Results and Discussion

### 1: Molecular Field Topology Analysis

Several structural motifs present in the compounds under study were found to occur in natural compounds. For example, dimethylallyl, isobutenyl, and similar groups in alkyl substituted amides **5a**-**5f'** are common building blocks of monoterpene alcohols, such as geraniol **7**, citronellol **8**, linalool **9**, α-terpineol, eugenol, which are all natural mosquito repellents and insecticides presumably as acetylcholinesterase (AChE) [65,66] and/or octopamine receptor [67,68] inhibitors.

Compounds **5q**-5**w** and **C40–C41** are benzamides and thus can be considered as DEET analogues. Structures **5b’-5d'** contain the cinnamamide **10** functional group, which possesses a diverse range of biological activity [69]. Functionally related cinnamyl acetate, cinnamaldehyde, and cinnamic alcohol demonstrate pronounced inhibitory effect against mosquito larvae, especially for *Ae* *. aegypti* [70], and also strong avian repellent activity [71]. Compounds from the **YF** family, comprising 2-amino- or 2-hydroxyethanol fragments, possibly mimic acetylcholine **11** and octopamine **12** neurotransmitters. Those containing ester and hydroxyallyl groups, resemble CO_2_, lactic acid **13**, and 1-octene-3-ol, which are the key food-related odors for mosquito females [72].

Compound **YF24**, 2-phenylcyclohexanol, was discovered in 1945 [73] and was used as a repellent until displaced by DEET. The hydroxyl and the phenyl groups of **YF24** resemble the structure of natural terpenoids, but **YF24** is less prone to oxidation and photochemical degradation due to the aromatic moiety present instead of linearly conjugated double bonds.

#### 1.1: MFTA Model

**Figure 4:**
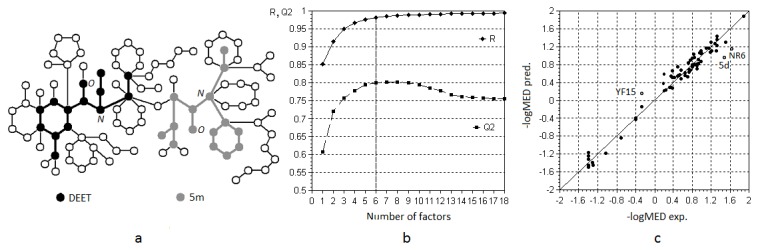
MFTA model: (a) molecular super-graph, (b) factor dynamics, and (c) fit plot. (a) The molecular supergraph is shown with two superimposed structures: DEET and *N*-cyclohexyl-*N*-ethyl-3-methylbutanamide (5m). The manner in which structures appear on MSG depends on how they can be superimposed onto the MSG as a whole. (b) The plot displays the change in correlation coefficient (R) and squared cross-validation coefficient (Q^2^) change as the number of factors changes. The best model is the one with the minimum possible number of factors and with R and Q^2^ at their highest values.

The best structure-activity model for the entire data set of 71 structures was obtained with the following molecular fields included: Gasteiger-Marsili atomic charges, van der Waals radii, local atomic lipophilicities, and hydrogen bond acceptor/donor scales. The molecular super-graph (MSG) image, a factor dynamics plot, and an “observed *vs.* predicted” fit plot are shown in Figure 4. As can be seen, superimposition of the representative structures **DEET** and **5m** onto the supergraph clearly indicates positions of the amide groups. The images of MSG with some other superimposed structures are given in Figure S1. According to the factor dynamic plot, the correlation coefficient *R* ^2^ and cross-validation *Q* ^2^ reach maximum values when six orthogonal components, or factors are taken for analysis, which is a reasonable number of independent variables for a training set of 71 compounds. The *R* ^2^ and Q_10%_^2^ values of 0.964 and 0.801, respectively, are high in any regard. As can be seen from the correlation fit plot, the data points form two loose clusters of relatively highly active and relatively less active compounds in the upper right and bottom left quadrants, but the data are generally distributed rather evenly over more than two logarithmic units. The high statistical quality of the QSAR model built for this structurally diverse data set attests its explanatory power and, with a good degree of accuracy, its capability to be used for prediction purposes.

#### 1.2: MFTA Interpretation

**Figure 5:**
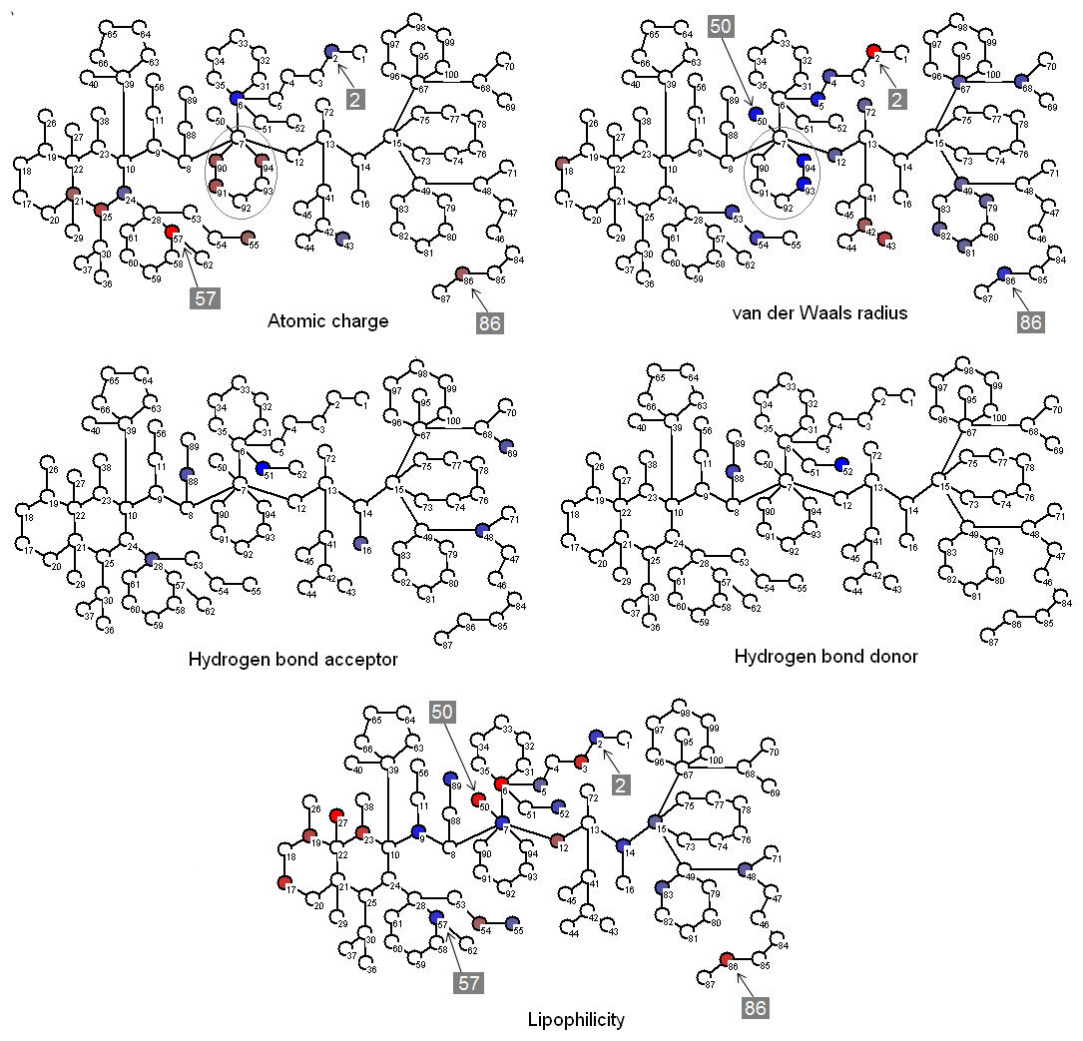
Visualization of relative contributions of molecular fields to the title activity. Positions discussed in the text are marked with arrows and boxed numbers; the six-membered ring (vertices 7 and 90-94) is encircled for clarity.

Analysis is facilitated by the color-coded schemes shown in Figure 5. Here, red (blue) color indicates an increase (decrease) in the activity as the descriptor value increases (decreases). Normalized and weighted values of the molecular fields are mapped on the molecular supergraph, with different shades of red and blue representing varying contributions, or impacts. These color-coded schemes are given in Figure 5 for the atomic charge, van der Waals radii, hydrogen bond acceptor, hydrogen bond donor, and lipophilicity descriptors. Positions color-marked in more than one molecular field are the most informative ones. For example, a decrease in atomic charge and hydrophobicity coupled with an increase in van der Waals radius of the atom in position 2 leads to an increase in the antilog MED value (which corresponds to the desired minimization of MED). As it follows from the descriptor values given in Table 3, for the atoms occupying position 2, the terminal CH_3_ group is preferable both over the -CH_2_- group and a vacancy. This can be found useful for inferring an optimal length of alkyl chains. Similar analysis and conclusions can be made for position 86.

**Table 3:** Descriptor values for selected positions of the supergraph.

Position	Atom	Q	vdW	Lipo	Position	Atom	Q	vdW	Lipo	HBa	HBd
2	C(-CH_2_-)	-0.0377	1.7	-0.3998	51	**C(-CH_2_-)**				**-2.0**	
	**C(-CH_3_)**	**-0.0428**	**1.7**	**-0.6327**		C(≡CH)				0.3	
	v.	0	0	-0.25	52	**C(-CH_3_)**					**-2.0**
50	**O_sp2_**		**1.5**	**-0.173**		O(-OH)					1.2
	C(-CH_3_)		1.7	-0.633	86	C(-CH_2_-)	-0.0377	1.7	-0.6327		
57	C(-CH_2_-)	-0.0334		-0.3998		**C(-CH_3_)**	-0.0428	1.7	-0.3998		
	**C(-CH_2_-OH)**	**0.0288**		**-0.9463**		**v.**	**0**	**0**	**-0.25**		

Most favourable atom types in terms of descriptor values are marked with bold (v. stands for vacancy)

The relatively smaller and more hydrophilic carbonyl oxygen atom in position 50 turns out to be more favorable from the steric (blue in van der Waals field) and lipophilicity (marked in red) points of view than the sp^3^ carbon atom, which reflects the much higher activity of **YF4** over **YF12**, **C42**, and **C43**. Position 57 highlighted in red in the electrostatic field and blue in the lipophilic field has optimal descriptor values for the carbon atom adjacent to a hydroxyl group, which can be related to a high repellent activity of **YF24**.

The color characteristics for positions 90, 91, 92, 93, and 94 clearly indicate that benzyl or cyclohexyl ring in this location is unfavorable in terms of both atomic charges and van der Waals radii; this is evidenced by a dramatic decrease in repellent activity of compounds **5b**’-**5i**'. The impact of hydrogen bond acceptor and donor parameters is less important, in line with the smaller number of colored nodes. The bright blue at positions 51 and 52 in hydrogen bond acceptor and hydrogen bond donor maps, respectively, suggests that the presence of strong hydrogen bond acceptor and donor in these positions results in a deterioration of the target activity.

The color scheme analysis helps identify molecular determinants of repellency and also serves as a good starting point to computer-aided design of novel repellents, which are to be identified beyond the existing training set.

### 2: Molecular docking

At the present time, a few X-ray crystal and solution NMR structures of insect OBP1 have been solved: 3N7H, 3K1E, 3R1O, 3R1P, 3R1V, and 2L2C [74]. In 3N7H, DEET taken as a reference binds at the interface formed by two OBP1 monomeric units. Interestingly, it does not form any direct hydrogen bonds with the protein. The only directional polar interaction at this site is the hydrogen bond between the carbonyl oxygen of DEET and the structural water molecule which also forms two additional hydrogen bonds with the Trp114 indole NH and the backbone carbonyl of Cys95. On the contrary, based on our expanded binding site docking results against the 3K1E OBP1 structure, the major part of the hit expansion compounds consistently dock in the middle of the OBP1 channel (occupied by PEG in the 3K1E structure) in the proximity of Phe123 and form hydrogen bonds to the backbone of either C=O or NH groups (or both) of Phe123. It was generally observed that the docked molecules were ranked higher if located deep inside the protein cavity (similarly to the PEG molecule in 3N7H). This can be explained by a larger van der Waals contact surface between the protein and the ligand, which leads to more favorable interactions and, accordingly, to higher docking scores.

Due to the mostly hydrophobic nature of protein-ligand interactions and a possibility of a conformational change upon binding, it is rather difficult to explain subtle differences in activity. Generally, the binding affinity is defined by shape complementarity between the protein and ligand. Very small compounds, such as **YF15**, may be binding at the binding site in several locations with partial occupancies. The high activity of **YF19** and **YF24** can be explained by the similarity of their shapes to DEET. Molecular volumes and shapes of these molecules are very close to each other, and they are located in the same spatial region.

A plausible pharmacophore scheme would include two features: (i) a hydrogen bond to backbone NH or carbonyl of Phe123; (ii) a deep-inside aromatic or hydrophobic moiety (e.g., corresponding to tolyl in DEET) bound in the hydrophobic pocket formed by a set of the aromatic and hydrophobic residues Phe59, Leu76, Trp114, Tyr122, and Phe123.

**Figure 6:**
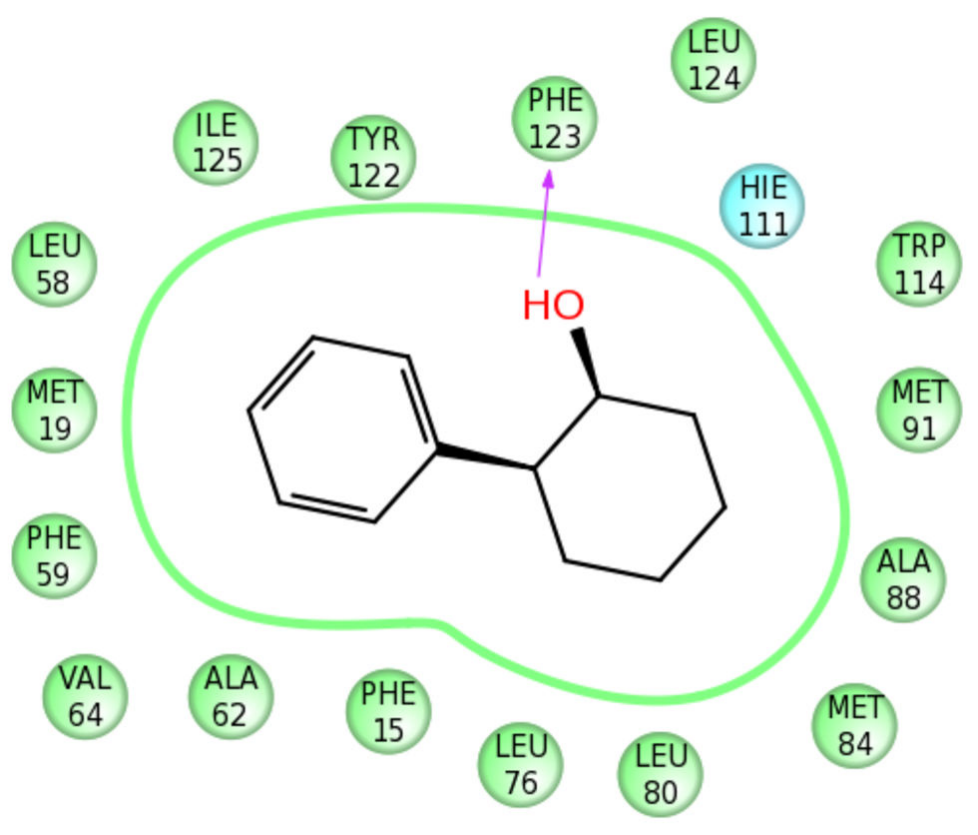
2D predicted binding mode diagram for YF24 ((1S,2S)-2-phenylcyclohexanol). 2D protein-ligand interaction diagram generated using the Ligand Interaction script in Maestro (Schrödinger Inc., www.schrodinger.com). It outlines a highly hydrophobic cavity consisted of a number of proximate hydrophobic residues (shown in green circles) where **YF24** binds. **YF24** is represented as a 2D chemical sketch. A hydrogen bond between the ligand and Phe123 is shown by an arrow.

**Figure 7:**
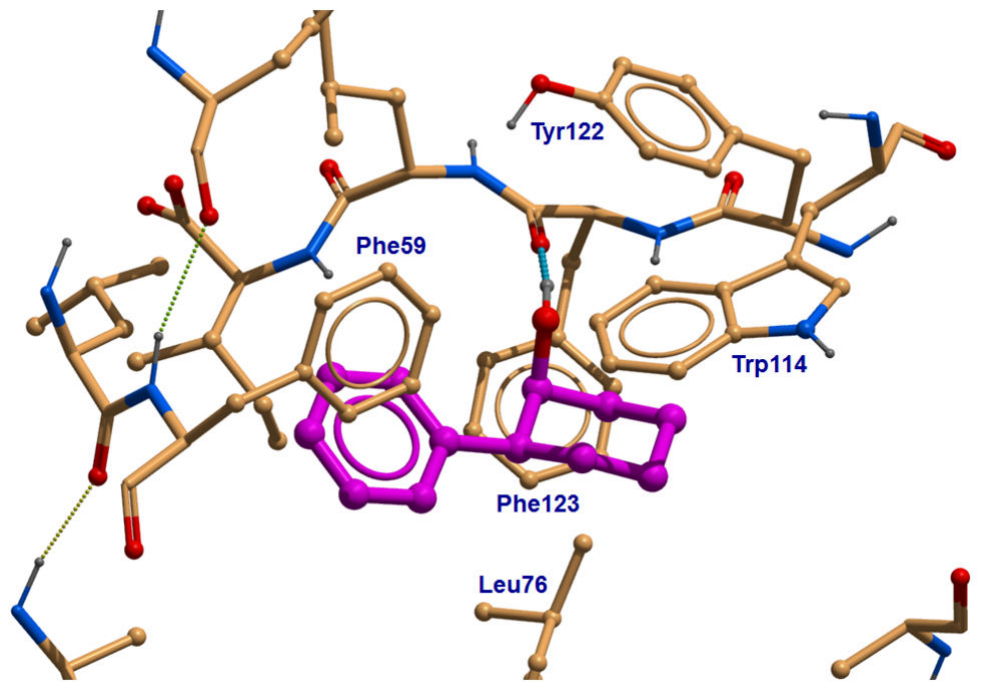
3D predicted binding mode for YF24 ((1S,2S)-2-phenylcyclohexanol). (**B**) Atomic details on how **YF24** binds to OBP1, as depicted by ICM Browser (MolSoft, www.molsoft.com). An anchoring interaction that defines the position and orientation of the ligand is the hydrogen bond between the hydroxyl-group of **YF24** and the backbone carbonyl group of Phe123. The rest of interaction is driven by a set of aromatic and hydrophobic residues, Phe59, Leu76, Trp114, Tyr122 and Phe123, that accommodates the cyclohexylbenzene core. Only proximate residues making contacts with **YF24** are shown.

One of the best of the docked structures in terms of the Glide score, **YF24**, is shown in Figures 6-7. The (1S,2S)-2-phenylcyclohexanol enantiomer is expected to have the highest binding affinity to OBP1 and also has the highest Glide SP score of -8.52. Other enantiomers have minor steric clashes, more strained geometry, and accordingly lower Glide scores (-8.39, -8.37, -8.16). It is clear that the molecule fits well inside the OBP1 cavity and also can form a strong hydrogen bond by its hydroxyl group with the backbone carbonyl of Phe123. Although purely computational, the docking results can serve as a rough, but still beneficial guidance for rational design. If a molecule fits poorly within the binding site in an *in silico* model, it is highly probable that its physiological effect will be negligible (if any).

Comparison of experimental activity with the docking score and binding interaction energies has not been straightforward in the field of computer-aided molecular design. One should be aware about the limitations and applicability ranges of the theoretical models implemented in the docking software. From examination of data in Table 4, it is apparent that the structure with the second best score has the highest repellent activity (lowest minimum effective dosage). In comparison, the activity of the compound was scored best by Glide is inferior in its experimental performance. The fact that the group of compounds that were least active in terms of MED (**5i**', **C40** and their analogues) are predicted to have similarly high docking scores is an interesting anomalous result. One plausible explanation is that although these compounds have a high potential to be tightly bound inside OBP1, they may occur completely inactive on the second step of the odorant transduction, i.e. upon the interaction with and stimulation of the odorant receptors (OR).

**Table 4:** Comparison of best SP Glide docking results with experimental MED.

**Compound**	**MED_obs_**	**Glide SP Score**
YF23	0.625	-8.520
**YF24**	0.039	-8.491
C40	25.00	-8.367
**YF19**	0.065	-8.363
5i'	20.500	-8.334
5w	5.160	-8.273
**YF25**	0.078	-8.210
C41	25.00	-7.640
**5h**	0.102	-7.508
**5l'**	0.140	-7.394
**YF21**	0.208	-7.226
5d'	20.250	-7.222
**5m**	0.172	-7.203
5b'	10.750	-7.019
5f	0.625	-6.944

An interesting observation comes from correlation of MED_obs_ and Glide SP scores for a subset of seven most active compounds (highlighted bold in Table 4). These structures are all compact, contain a hydroxyl or carboxyl oxygen, and can be matched to either a biphenyl or naphthalene scaffold. The high correlation coefficient of 0.922 suggests that a possible chemotype can exist that binds strongly to *Aaeg*OBP1 and also acts as a strong repellent. This provides for the first time a direct link between the predicted ligand-OBP interaction energy and insect behavioral response. This relationship between OBP binding and repellency is not general of course, because in the olfactory cascade OBP is just a gateway and it is believed that the odorant receptors may play the major role. Table 4 provides examples of compounds with high affinity to OBP which are nevertheless inactive as repellents.

### 3: Bioassay of hit expansion compounds

The hit expansion procedure accomplished through the eMolecules Plus database resulted in 344 commercially available analogues of the four starting scaffolds. Based on the Glide SP docking of these analogues against the 3K1E OBP1 structure and thorough visual inspection, we have identified 36 compounds that looked promising as potential OBP1 ligands.

**Figure 8:**
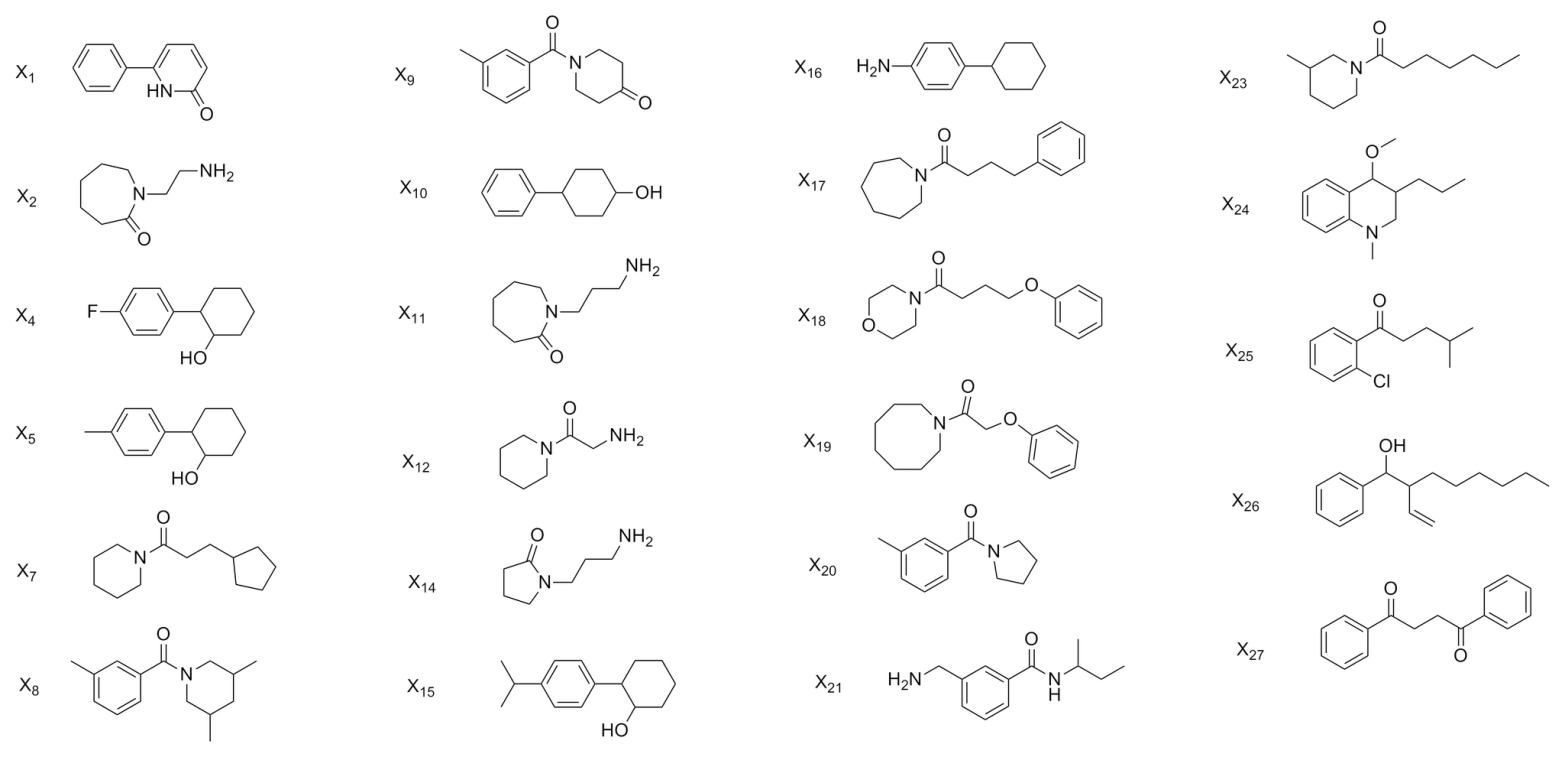
Chemical structures of test set compounds.

The repellency bioassay described in the Materials and Methods section was carried out to determine the MED values for 27 out of 36 compounds from the hit expansion dataset. These experimental MED values pertinent to the hit extension dataset (designated **X1** to **X27**, where **X** stands for eXpansion) are in Table 5 (with the chemical structures displayed in Figure 8) along with the MFTA predictions and the Glide scores. Two of the identified compounds, **X4** and **X23**, exhibit very high repellency. **X4** is structurally similar to 2-phenylcyclohexanol (**YF24**), which has been known to be a repellent since 1945, but only methyl substituted analogues of it were studied [75]. **X4** is therefore a successful expansion of the existing scaffold, and its repellent activity is reported here for the first time. **X23** was first identified as a repellent in 1975 by McGovern et al. [76], but no effective dosage was reported in that study. Here, we report a minimum effective dosage of 0.039 µg/cm^2^; this is repellent activity comparable to that of DEET.

**Table 5:** Comparison of Glide SP scoring, MFTA predicted MED values, and bioassay results for selected compounds.

Compound	Glide score	MED_pred_	MED_obs_
X1	-9.109	0.307	>2.5^a^
X^2^	-8.166	-	>2.5
X4	-8.715	0.030	0.078
X5	-8.643	0.067	0.156
X7	-7.797	0.112	0.417
X8	-	-	>2.5
X9	-8.106	0.182	>2.5
X10	-	0.330	1.25
X11	-7.910	-	>2.5
X12	-7.757	0.096	>2.5
X14	-6.684	-	>2.5
X15	-	-	>2.5
X16	-8.405	0.303	0.261
X17	-	0.082	1.667
X18	-	0.219	>2.5
X19	-	-	>2.5
X20	-7.666	-	>2.5
X21	-7.529	2.786	>2.5
X23	-6.832	0.061	0.039
X24	-	0.036	>2.5
X25	-	0.198	1.87
X26	-	0.010	>2.5
X27	-	-	>2.5

a repellent activity was not observed at the highest dosage of 2.5 µg/cm^2^

The minimum effective dosages of **X4** and **X23** are slightly higher than that of **YF24**, and this observation leads to the conclusion that 2-phenylcyclohexanol is a viable scaffold for developing more diverse active repellent compounds. It is a matter of further computational and experimental research as to how to modify the structure of **YF24** to increase the repellent activity. But at least one structural feature is made clear: substituents in the *para*-position of the 2-phenylcyclohexanol ring are not favorable. Fluorine substitution in this position results in a slight decrease in activity, while methyl decreases the activity by a factor of two compared to fluorine. Based on this observation, the introduction of additional substituents to the cyclohexyl ring may become favorable. Hydroxyl and other polar groups such as nitro, nitrile, or halogen would be beneficial structural elements. This structure-activity work is in progress.

Although the quantitative correspondence between the MFTA predicted and observed MED values is not perfect, the qualitative trend is satisfactory. As evident in Table 5, MFTA predicts very low values for **X4** and **X23**, whereas for the other compounds the MFTA predictions are an order of magnitude larger. The docking score is in qualitative agreement with the experimental data despite the very favorable docking scores assigned to inactive compounds **X1** and **X2**. This mismatch has an important implication which reiterates what was already mentioned above; specifically, not every compound having a high affinity to OBP1 is a good repellent. The mosquito olfactory mechanism is complex, and OBP1 is most likely just the first step in developing a response. OBP1 either brings the odorant molecule into a direct contact with the olfactory receptors or exerts an allosteric action upon the OR. In both cases, the initial binding state of the *Aaeg*OBP1-odorant complex can change dramatically as the recognition event proceeds. It is quite reasonable to assume that the compounds sharing the 2-phenylcyclohexanol scaffold are bound in such a favorable mode that they are able to activate the OR machinery.

## Conclusions

Analysis by QSAR revealed molecular determinants of repellent action against *Aedes aegypti*, and this knowledge was translated into search queries for a scaffold hopping step. Molecular docking with Glide against the *Aaeg*OBP1 3D structure helped identify highly promising scaffolds and individual compounds possessing mosquito repellent activity. The computational findings were confirmed by behavioral bioassay with *Aedes aegypti* mosquito species, the vector for yellow fever, chikungunya, and dengue fever. Complexation of an odorant molecule with the odor-binding protein OBP1 has been demonstrated to be a significant, although not general step in the development of mosquito repelling response. The important role of the hydroxyl group in the cyclohexanol scaffold has been verified by both QSAR and molecular docking. Integration of computational and experimental approaches for the first time proposed in this study exemplifies a genuine computer-aided discovery of mosquito repellents.

## Supporting Information

Figure S1 Examples of structure superimposition on the molecular supergraph for selected compounds from the training dataset (Tables 1 and 2 of the paper). (TIF)
