# Correction: Promising *Aedes aegypti* Repellent Chemotypes Identified through Integrated QSAR, Virtual Screening, Synthesis, and Bioassay

**DOI:** 10.1371/annotation/1dc83f26-8045-4b63-8a86-24c7b71a7f79

**Published:** 2014-01-06

**Authors:** Polina V. Oliferenko, Alexander A. Oliferenko, Gennadiy I. Poda, Dmitry I. Osolodkin, Girinath G. Pillai, Ulrich R. Bernier, Maia Tsikolia, Natasha M. Agramonte, Gary G. Clark, Kenneth J. Linthicum, Alan R. Katritzky

Reference 46 was cited incorrectly. The corrected reference is:

Tsitsanou KE, Thireou T, Drakou CE, Koussis K, Keramioti MV, Leonidas DD, Eliopoulos E, Iatrou K, Zographos SE (2012)

Anopheles gambiae odorant binding protein crystal complex with the synthetic repellent DEET: implications for structure-based design of novel mosquito repellents. Cellular and Molecular Life Sciences 69(2): 283-297. doi:10.1007/s00018-011-0745-z. PubMed: 21671117.

An incorrect reference appears in the Materials and Methods section, in the second to last sentence of the first paragraph under '4: Hit Expansion and Molecular docking.' The corrected sentence should read: "Despite the fact that the Anopheles gambiae OBP1 structure (PDB 3N7H, 1.6 Å) [46] was solved at a higher resolution, we selected the Aedes aegypti 3K1E structure for docking because of our interest in this particular mosquito species."

