# Supplementary figures and images for: Promising Aedes aegypti Repellent Chemotypes Identified through Integrated QSAR, Virtual Screening, Synthesis, and Bioassay
Source: PLoS One. 2013 Sep 6;8(9):e64547. doi: 10.1371/journal.pone.0064547 (PMC3765160; doi:10.1371/journal.pone.0064547)

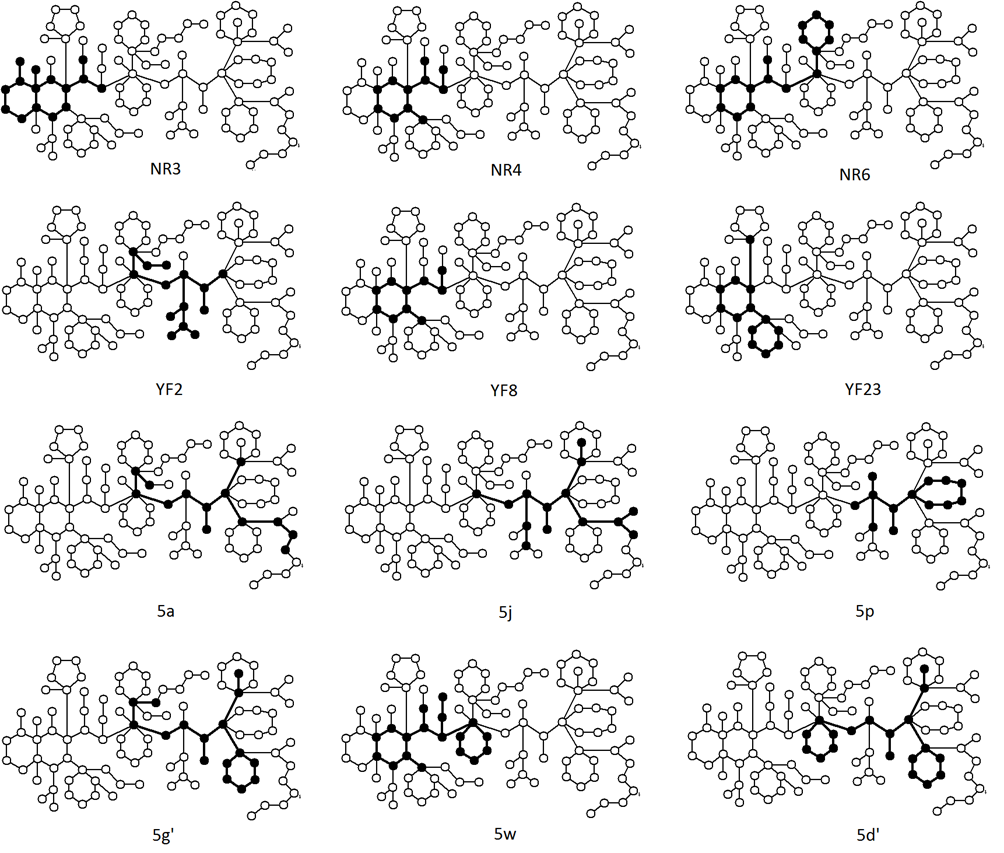

Supplement: Figure S1 — (TIF) [file pone.0064547.s001.tif]
